# Gut Microbiota Composition in Patients with Neurodegenerative Disorders (Parkinson’s and Alzheimer’s) and Healthy Controls: A Systematic Review

**DOI:** 10.3390/nu15204365

**Published:** 2023-10-13

**Authors:** Fatemah Sadeghpour Heravi, Kaveh Naseri, Honghua Hu

**Affiliations:** 1 Macquarie Medical School, Macquarie University, Sydney, NSW 2109, Australia; 2 School of Health and Biomedical Sciences, RMIT University, Melbourne, VIC 3983, Australia; naserikaveh1@gmail.com; 3 Innovation Center of Translational Pharmacy, Jinhua Institute of Zhejiang University, Jinhua 321016, China

**Keywords:** gut microbiota, Parkinson’s disease, Alzheimer’s disease, gut–brain axis, dysbiosis

## Abstract

This systematic review aims to provide a comprehensive understanding of the current literature regarding gut microbiota composition in patients with Parkinson’s disease (PD) and Alzheimer’s disease (AD) compared to healthy controls. To identify the relevant studies, a thorough search of PubMed, Medline, and Embase was conducted following the PRISMA guidelines. Out of 5627 articles, 73 studies were assessed for full-text eligibility, which led to the inclusion of 42 studies (26 PD and 16 AD studies). The risk of bias assessment showed a medium risk in 32 studies (20 PD studies and 12 AD studies), a low risk in 9 studies (5 PD studies and 4 AD studies), and 1 PD study with a high risk. Among the PD studies, 22 out of 26 studies reported a different gut microbiota composition between the PD cases and the healthy controls, and 15 out of 16 AD studies reported differences in gut microbiota composition between the AD cases and the healthy controls. The PD and AD studies consistently identified the phyla Bacteroidetes, Firmicutes, and Proteobacteria as prevalent in the gut microbiota in both the healthy groups and the case groups. Microbial dysbiosis was specifically characterized in the PD studies by a high abundance of *Akkermansia*, *Verrucomicrobiaceae*, *Lachnospiraceae*, and *Ruminococcaceae* in the cases and a high abundance of *Blautia*, *Coprococcus*, *Prevotellaceae*, and *Roseburia* in the controls. Similarly, Bacteroides and Acidobacteriota were abundant in the AD cases, and *Acidaminococcaceae*, Firmicutes, *Lachnospiraceae*, and *Ruminiclostridium* were abundant in the AD controls. The microbial signature assessment showed the association of several microbial taxa, including *Akkermansia*, *Lachnospiraceae*, *Verrucomicrobiaceae*, *Bifidobacterium*, *Ruminococcacea*, and Verrucomicrobia with PD and *Ruminococcaceae*, *Bacteroides*, and Actinobacteria with AD. The microbial diversity evaluations in the PD and AD studies indicated comparable alpha diversity in some groups and distinct gut microbiota composition in others, with consistent beta diversity differences between the cases and the controls across multiple studies. The bacterial signatures identified in this study that are associated with PD and AD may offer promising prospects for efficient management and treatment approaches.

## 1. Introduction

Neurodegenerative disorders, such as Parkinson’s disease (PD) and Alzheimer’s disease (AD), pose significant challenges to healthcare systems and influence people’s cognitive and physical abilities. PD is the second most common neurodegenerative disorder after AD, affecting approximately 1–2% of the population over the age of 65 [1]. Also, AD is the most common cause of dementia worldwide, accounting for 60–70% of all cases [2]. It is estimated that AD affects more than 50 million people globally, and this number is expected to triple by 2050 [3].

Despite the differences in clinical presentation and pathology, PD and AD share common features such as progressive neurodegeneration, cognitive decline, and decreased motor function [4,5]. The etiology of these disorders remains complex and multifactorial, involving a combination of genetic and environmental factors [6]. Recent findings suggest that the gut microbiota, a diverse community of microorganisms found in the gastrointestinal tract, may play a crucial role in the pathophysiology and development of neurodegenerative disorders [7].

The gut–brain axis, a bidirectional communication system between the gut and the central nervous system, is actively influenced by the gut microbiota and its metabolites. This axis represents a complex network through which the gut and brain can influence each other’s functions and activities [8]. Imbalanced gut microbiota can impact the brain function through various mechanisms, including: (a) activation of pro-inflammatory responses; (b) production of neuroactive compounds, such as short-chain fatty acids (SCFAs) like acetate, propionate, and butyrate; (c) initiation of immune responses triggered by microbial metabolites, such as lipopolysaccharides (LPS); (d) contribution to the formation and accumulation of abnormal brain proteins, such as amyloid; (e) disruption of neurotransmitter production in the gut (e.g., serotonin and dopamine) [9,10]; and (f) disruption of gastrointestinal integrity, leading to microbial translocation into the bloodstream and brain, as well as dysfunction of blood–brain barrier integrity [11,12].

Decreased levels of SCFAs as a result of a lesser abundance of a beneficial microbial population have been linked to intestinal barrier malfunction and neuroinflammation and to an increase in the susceptibility of neurons to injury in AD and PD cases [13,14,15]. Additionally, increased levels of LPS-producing bacteria (such as *Baeroides*) have been linked to the translocation of microbial substances into the brain and the promotion of neuroinflammation, a crucial pathological feature associated with a variety of neurological disorders, including AD and PD [16,17,18].

Studies have shown that an increased abundance of the *Verrucomicrobiaceae* family, particularly the *Akkermansia* genus (*A. muciniphila*) has been linked to increased mucin degradation in patients with PD and AD [17,18,19]. Mucin-degrading bacteria can destroy the protective mucus layer in the gut and lead to compromised gut barrier integrity, potentially contributing to increased permeability and the translocation of harmful substances into the bloodstream, triggering neuroinflammatory responses in PD and AD [17,20].

A decreased abundance of SCFA-producing bacteria, such as *Faecalibacterium prausnitzii*, *Roseburia*, and *Coprococcus*, has also been observed in PD cases, which may explain the altered gut barrier function and the increased inflammation observed in PD individuals [15]. 

Studies have also shown a decrease in beneficial bacteria such as *Prevotellaceae* and *Lachnospiraceae* [21,22,23] and reduced levels of SCFA producers [24], along with an increase in potentially pro-inflammatory bacteria such as *Enterobacteriaceae* in PD and AD cases [25,26,27,28]. Bardenhorst et al.’s study highlighted the significance of reduced abundance in butyrate-producing microbial communities like *Roseburia* and *Faecalibacterium*, as well as the increased mucus degradation activity by *Akkermansia*. These factors were associated with the promotion of intestinal inflammation, leaky gut, and the subsequent translocation of microbes and metabolites from the gut into the bloodstream and enteric nervous system in PD cases [29]. Another study showed the increasing trend of Proteobacteria and the decreased abundance of Firmicutes and *Bifidobacteria* in PD and AD cases [30]. 

Also, a recent systematic review on 11 studies showed lower microbial diversity in AD cases compared to healthy controls, with a high abundance of Proteobacteria, *Bifidobacterium*, and *Phascolarctobacterium* in AD cases. However, this study only included studies from the USA and China; this can significantly influence the interpretation of the gut microbiota findings [31]. 

Also, it is worth pointing out that in recent years, an emerging body of research has shown the complex association between nutrition, gut microbiota composition, and the development of several diseases in humans. For instance, studies have shown that adhering to a Mediterranean diet rich in fruits [32], vegetables [33], and omega-3 fatty acids [34] has been associated with a more diverse and beneficial microbial profile. Similarly, a diet high in dietary fiber can promote the growth of beneficial bacteria in the gut, reducing inflammation and potentially controlling the risk of neurodegenerative diseases [35]. These findings underscore the importance of dietary factors as a crucial component of the complex interplay between the gut microbiota and the development of AD and PD.

Although previous studies have provided valuable insights into gut microbiota composition, there remains a need to comprehensively assess and synthesize the existing body of knowledge in this area. This systematic review paper addresses this gap by presenting a rigorous evaluation of gut microbiota composition in patients with PD and AD, as well as in healthy controls. In contrast to prior research that may have been limited to specific timeframes or geographical locations, we performed an inclusive approach to include all the relevant studies available to date. This strategy enables us to not only consolidate previous findings but also to address the potential limitations identified in some of the previous research. Additionally, this review summarized bacterial communities based on the frequency with which they have been reported across the studies, thereby shedding light on the most consistently observed microbial communities. Through a thorough assessment of the risk of bias, adherence to the PRISMA guidelines, and the identification of the potential microbial signatures associated with these neurodegenerative disorders, this paper provides a deeper understanding of gut microbiota in both the PD and the AD cases and controls. Furthermore, we not only highlight the limitations identified by the original authors but also introduce additional unaddressed limitations, along with strategies to overcome them to increase the reliability and comparability of microbiota findings.

Understanding the link between gut microbiota composition and neurodegenerative disorders is critical as it may provide insights into disease etiology, progression, and potential therapeutic targets. Therefore, conducting a systematic review of the existing literature on gut microbiota composition in patients with PD and AD is vital to evaluate and synthesize the current evidence.

This systematic review aims to comprehensively evaluate the available literature on gut microbiota composition in patients with PD and AD, focusing on microbial alterations and their potential implications in disease pathogenesis. By providing details on the relationship between gut microbiota and neurodegenerative disorders, this review can potentially contribute to the development of novel strategies for diagnosis, prevention, and treatment through gut microbiota modulation.

## 2. Methodology

This review was conducted under the guidelines provided by the preferred reporting items for systematic reviews and meta-analyses (PRISMA) [36]. The protocol for this review was registered prospectively with PROSPERO under the registration number CRD42023422561.

### 2.1. Searching Strategy

Relevant research databases (PubMed, Medline, and Embase) were thoroughly searched from the beginning to 11 July 2023. The search was conducted using a combination of keywords along with various logical operators (AND, OR, (), “”). The keywords used included “microbiome”, “microbiota”, “intestinal flora”, “intestinal microbi”, “dysbiosis”, and “gut microbio*” combined with “Parkinson’s Disease” and “Alzheimer’s Disease”. The complete search strategy can be found in Supplementary Material S1. No specific restrictions regarding time, location, or study design were applied.

### 2.2. Selection Criteria and Screening

The studies were uploaded to Covidence (www.covidence.org, 1 May 2023) for eligibility screening by two authors (FSH, KN). Conflicts were resolved through discussion between the authors. The initial screening involved assessing the title and abstract, and further screening was conducted based on the full text of the studies. Inclusion (A) and exclusion criteria (B) were applied to determine the eligibility of the studies:

A: (i) Human studies; (ii) studies comparing the composition of gut microbiota between healthy individuals and patients with Parkinson’s or Alzheimer’s disease; (iii) English studies; and (iv) studies utilizing next-generation sequencing-based approaches to compare the gut microbiota composition between healthy individuals and patients with Alzheimer’s or Parkinson’s disease (such as 16S rRNA sequencing or shotgun metagenomics).

B: (i) Animal studies; (ii) studies without appropriate healthy and control groups; (iii) studies employed irrelevant methodology (such as culture-based or targeted PCR methods); and (iv) review articles.

### 2.3. Data Extraction

The data were extracted independently by two authors (FSH, KN) from the included studies. In the case of any conflicts, the authors resolved them through discussions. The following data were extracted whenever they were available: the year of publication, study location, recruitment period, population size, age range, gender, the total number of collected samples, number of samples per participant, sample type, inclusion and exclusion criteria, method of preserving samples before DNA extraction, the storage temperature of the samples, DNA extraction method, sequencing platform, specific regions of the 16S rRNA gene that were sequenced, primers, the total number of sequencing reads or sequencing reads per sample, availability of the data, reference database, significant findings related to gut microbiota, changes observed in the gut microbiota over time in both the healthy individuals and the cases, alpha (microbial diversity within individual samples) and beta diversity indices (microbial diversity between groups), methods utilized to determine differential abundance between the cases and the controls, and the limitations and strengths highlighted in the study.

### 2.4. Risk of Bias Assessment

Two authors (FSH, KN) conducted a risk of bias assessment, examining potential biases known to impact the findings across four main domains: (1) sampling, (2) comparability, (3) data reporting, and (4) outcome measurement biases. Each domain was also broken down into subdomains to assess the risk of bias with more details. A traffic light plot summarizing the risk of bias was generated using Robvis [37]. Additional information, including subdomains within each domain, can be found in Supplementary Material S2.

### 2.5. Evaluation of Findings

To comprehensively evaluate the gut microbiota composition in individuals with PD or AD, a narrative analysis approach was utilized. This involved conducting an extensive literature review to gather and compare relevant data on the core gut microbiota, prevalent microbial communities, comparisons of alpha and beta diversity in cases and controls, and bacterial taxa associated with PD and AD.

## 3. Results

### 3.1. Study Selection

**Figure 1:**
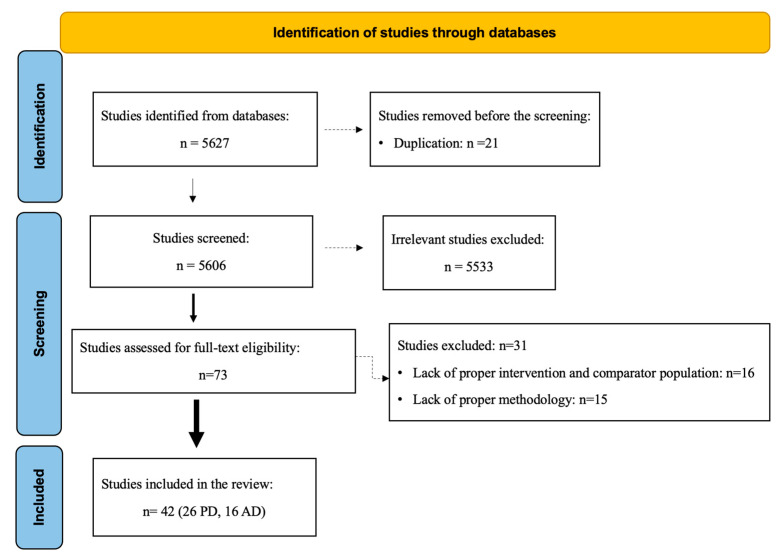
PRISMA flow diagram of the literature search and article selection.

Through the initial database search, a total of 5627 articles were identified. After removing duplicate records (*n* = 21), 5606 unique records remained. Following the title and abstract screening, 5533 records were identified as irrelevant. The remaining 73 records underwent full-text screening to determine eligibility. Among these, 31 studies were excluded due to the absence of appropriate case and control groups (*n* = 16) or inappropriate methodology (such as culture-based or targeted PCR studies) (*n* = 15). Ultimately, a total of 42 eligible studies (26 focused on Parkinson’s disease and 16 on Alzheimer’s disease) were included in this review (Figure 1).

### 3.2. Risk of Bias Assessment 

**Figure 2:**
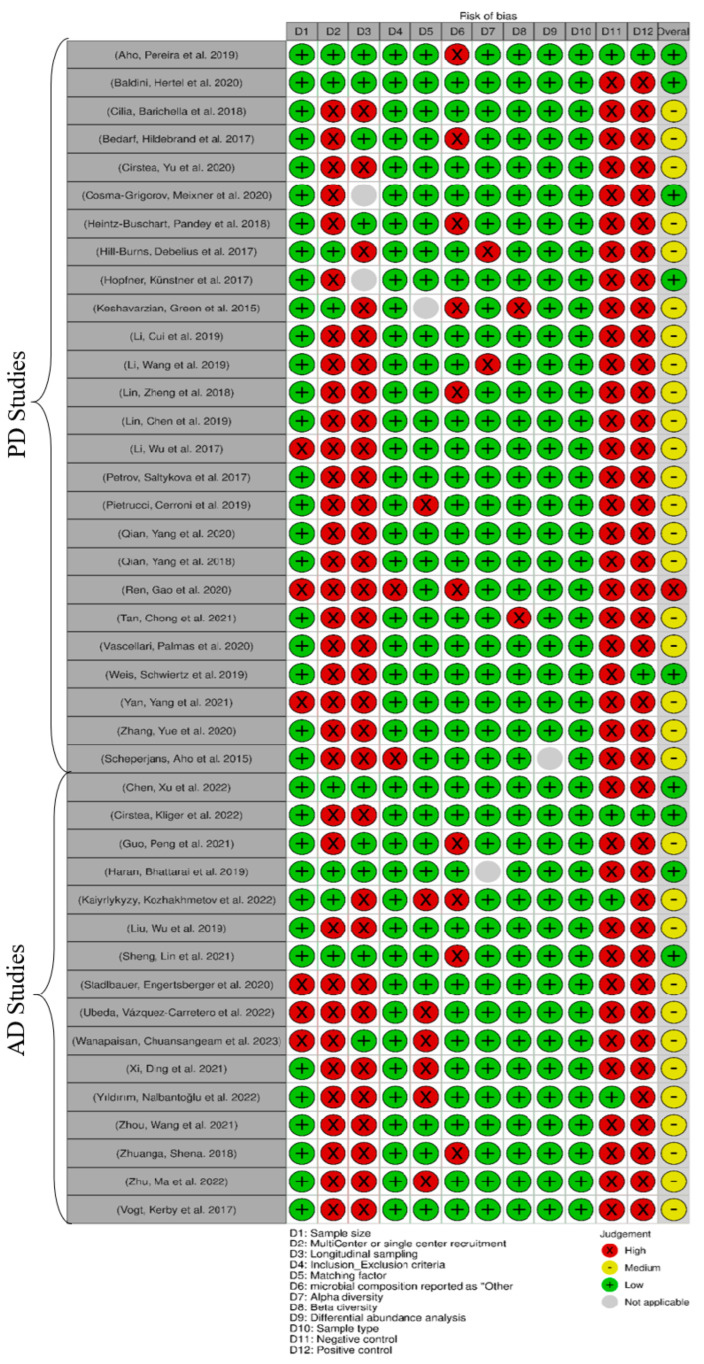
Risk of bias assessment with corresponding biases (D1 to D12) and risk indicators: green: low risk; yellow: medium risk; red: high risk; and gray: not applicable [14,17,18,19,20,21,22,23,24,25,26,27,28,38,39,40,41,42,43,44,45,46,47,48,49,50,51,52,53,54,55,56,57,58,59,60,61,62,63,64,65,66].

Of the articles, nine studies had the least risk of bias (five PD studies and four AD studies) [17,22,26,38,39,40,41,42,43], thirty-two studies had a medium risk (twenty PD studies and twelve AD studies) [14,18,19,20,21,23,24,25,27,28,44,45,46,47,48,49,50,51,52,53,54,55,56,57,58,59,60,61,62,63,64,65], and one PD study had a high risk [66] (Figure 2). A lack of appropriate experimental controls was observed in 39 studies for positive control [14,17,18,19,20,21,22,23,24,25,27,28,39,40,41,42,44,45,46,47,49,50,51,52,53,54,55,56,57,58,59,60,61,62,63,64,65,66] and in 38 studies for negative control [14,17,18,19,20,21,22,23,24,25,27,28,39,40,41,42,43,44,45,47,48,49,50,51,52,53,54,55,56,57,58,59,60,62,63,64,65,66]. Furthermore, 34 studies mainly recruited participants from a single center [18,19,20,21,22,23,25,26,27,28,41,43,44,45,47,48,49,50,51,52,53,54,55,56,57,58,59,60,61,62,63,64,65,66], and 31 studies did not employ a longitudinal sampling method [14,18,21,23,24,25,26,27,28,43,44,46,47,48,49,50,51,52,53,54,55,56,57,59,60,61,62,63,64,65,66]. In 10 studies, the microbial composition was reported as “Other” without providing specific details [14,19,20,27,38,42,45,46,51,65,66]. Additionally, seven studies did not adequately address matching factors between the cases and the controls [18,46,51,58,59,61,64]. Also, the sample size was small in six studies (*n* < 49) [18,48,54,58,60,66]. Two studies did not specify their inclusion/exclusion criteria [25,66], while another two studies did not assess alpha [21,24] and beta diversity comparison [14,55], respectively (Supplementary Material S3).

### 3.3. General Characteristics of Included Studies 

The included studies were mainly published between the years 2014 and 2022; they were conducted in various locations, encompassing a diverse range of locations. China emerged as the leading contributor to this field, with 17 studies [21,27,28,40,42,45,47,48,52,53,59,60,62,63,64,65,66], followed by Germany (5 studies) [19,20,22,41,43] and the USA (4 studies) [14,17,24,57]. Other countries made smaller contributions. The study populations varied between 20 and 350 participants (median: 98), with 10 to 237 cases and 10 to 162 controls. Stool samples were consistently the most prevalent sample type utilized across the studies. 

DNA extraction predominantly relied on the QIAamp DNA Stool Mini Kit and PowerSoil DNA Isolation Kit. Among the sequencing platforms, Illumina MiSeq was the most commonly utilized platform and was utilized in 34 studies [18,20,21,22,23,24,26,28,38,39,40,41,42,43,44,45,46,49,50,51,53,54,55,56,57,58,59,60,61,63,64,65,66]. The V3–V4 region was the primary target region for sequencing in 22 studies [22,23,28,38,39,40,42,45,49,50,51,53,55,56,58,59,60,61,63,64,65,66]. Notably, the forward primers 341F1 (in 9 studies) [21,22,38,40,42,53,55,61,63] and 515F (in 7 studies) [18,20,24,26,44,47,66] and the reverse primers 806R (in 10 studies) [18,22,24,26,42,44,47,59,64,65] and 805R (in 6 studies) [20,21,40,55,61,63] were frequently used. 

In this manuscript, a summarized data extraction table is provided (Table 1); however, a comprehensive data extraction table, including all the extracted details, is available in Supplementary Material S4.

### 3.4. Gut Microbiota Comparison between Cases and Controls

Among the PD studies, 22 out of 26 studies reported a different gut microbiota composition between the PD cases and the healthy controls [19,20,21,22,23,24,25,38,39,44,47,48,49,50,51,52,53,55,56,60,62,66], supporting the association between PD and gut microbial alterations. Additionally, 15 out of 16 AD studies reported differences in gut microbiota composition between the AD cases and the healthy controls [17,18,28,40,42,45,46,54,57,58,59,61,63,64,65]. 

These studies utilized a variety of analytical methods, including OTU clustering, diversity analysis (richness and evenness measurements), taxonomic classification, and differential abundance analysis to compare the gut microbiota composition between cases and controls, as discussed further in this systematic review.

### 3.5. Core Gut Microbiota

The comparison of the PD studies has revealed a set of abundant bacteria that were consistently found in both the case and the control groups, regardless of the individuals’ health status.

Bacteroidetes (eight studies) [21,25,48,49,56,60,62,66], Firmicutes (six studies) [21,25,48,56,60,62], Ruminococcaceae (five studies) [19,20,21,27,38], Proteobacteria (four studies) [21,25,56,60], Lachnospiraceae [20,27,38], Actinobacteria [25,56,60], and Bacteroidaceae [19,21,27] (each in three studies), Verrucomicrobia (two studies) [25,56], Clostridiales order [19] and Fusobacteria [25] (each in one study) were identified as common microbial communities in both the PD healthy groups and the case groups, regardless of their grouping. 

Similarly, in the context of the AD studies, Bacteroidetes and Firmicutes (each in seven studies) [18,38,42,57,61,64,65] emerged as the most prevalent phyla. Proteobacteria (six studies) [38,42,57,61,64,65], Actinobacteria (five studies) [18,42,57,61,64], Verrucomicrobia (three studies) [40,57,61], and Fusobacteria (one study) [40] were also consistently identified in the AD studies as core gut microbiota in both the case and the control groups. 

**Figure 3:**
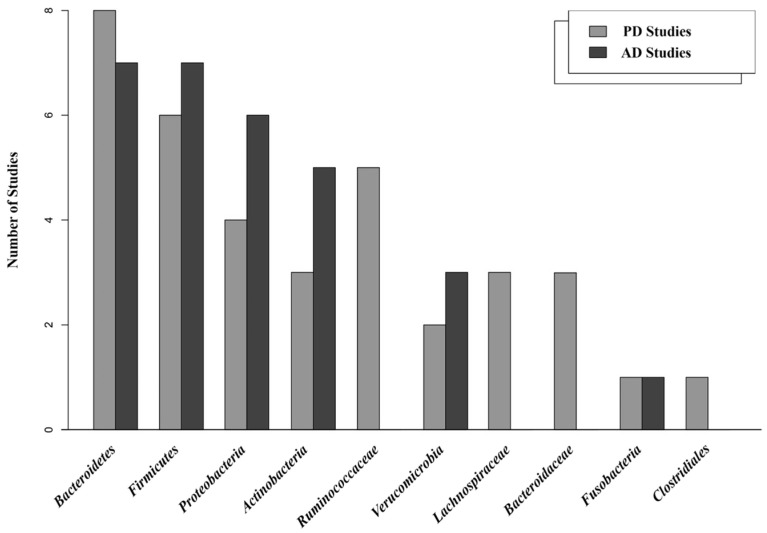
Predominant bacterial communities, at different taxonomic ranks, identified as core gut microbiota in PD and AD studies, irrespective of the participants’ health status.

As a result, both the PD and the AD studies consistently identified the phyla Bacteroidetes, Firmicutes, and Proteobacteria as prevalent in the gut microbiota in both the healthy and the case groups (Figure 3).

### 3.6. Microbial Communities in the Healthy Group

Several prevalent bacterial taxa have been consistently identified within specific taxonomic ranks in the healthy control groups in both the PD and the AD studies. In the PD studies, the genera Blautia (three studies) [14,38,41], Coprococcus (two studies) [14,41], and Roseburia (two studies) [41,46], as well as the family Prevotellaceae (two studies) [21,38] were consistently found. Additionally, other bacterial communities such as those within the orders Bacteroidales, the genus Bacteroides, the family Clostridieceae, and the genus Faecalibacterium, among others, were identified in individual studies.

Similarly, Acidaminococcaceae and Lachnospiraceae (each in two studies) [40,45] were identified as prevalent bacteria in healthy individuals in the AD studies. Furthermore, several other bacterial communities, such as the family Bacteroidaceae, the genus Agathobacter, and the species Bacteroides fragilis were observed in single studies.

### 3.7. Microbial Communities in the Case Group

The comparison of highly abundant bacteria in the PD and AD case groups revealed several key findings at specific taxonomic ranks. In the PD studies, the genus Akkermansia was consistently identified in 10 studies [14,20,21,23,24,44,47,49,52,56], with Akkermansia muciniphila reported in 3 studies [19,39,52], highlighting its potential significance in PD pathogenesis. Additionally, the family Verrucomicrobiaceae (eight studies) [19,20,21,23,24,27,47,56], the family Lachnospiraceae (six studies) [21,22,23,24,41,51], and the genus Bifidobacterium (five studies) [21,22,47,60,66] were found to be highly abundant in the PD cases. Similarly, in the AD studies, the genus Bacteroides [17,18,46,58,61] and the phylum Acidobacteriota [18,26,46,65] were consistently found to be prevalent in the AD cases. 

**Figure 4:**
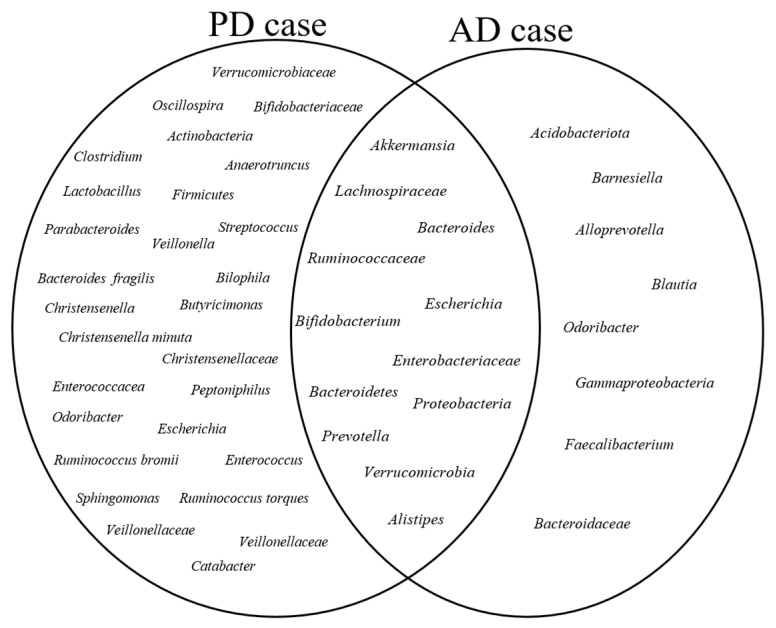
Highly abundant bacterial communities in PD and AD Cases: A Venn diagram representing bacterial communities reported as highly abundant in PD and AD cases across multiple included studies. The circles represent the bacterial taxa found in each disease group, while the overlapping sections denote the shared bacteria between PD and AD cases. These highly abundant microbial communities were consistently reported as such when compared to their respective healthy control groups in the included studies.

Other microbial communities reported in a lower number of studies in the AD and PD cases are shown in Figure 4.

Figure 4 shows increased microbial communities in the AD and PD cases. A Venn diagram illustrates the highly abundant bacterial communities shared between the PD and AD case groups. The AD and PD circles show specific bacterial taxa that are abundant in each disease, with the left circle representing the PD cases and the right circle representing the AD cases. These shared microbial communities, represented by the overlapping sections of the diagram, suggest a potential link between the shared microbial communities and the pathophysiology of PD and AD. 

Low abundant bacteria in the PD and AD cases provided further insights into the microbial composition of the gut microbiota in these groups. In the PD studies, the family Lachnospiraceae [14,23,24,27,44,51,56] and the genus Faecalibacterium [25,43,44,48,50,60] were identified in seven and six studies, respectively, suggesting their potential role as less abundant but still significant bacterial taxa in PD. 

Similarly, in the AD studies, the phylum Firmicutes was found in low abundance in the AD cases across six studies [18,26,28,42,57,61], followed by the phylum Bacteroidetes [26,28,61,65] and the family Lachnospiraceae [28,42,54,65] in four studies (Figure 4). A comprehensive overview of the list of bacteria identified in lower numbers of studies is provided in Supplementary Material S5.

### 3.8. Bacterial Diversity in PD and AD Studies (Alpha and Beta Diversity)

In the PD and AD studies, a diverse range of alpha (microbial diversity within individual samples) and beta diversity indices (microbial diversity between groups) were utilized to evaluate the diversity and composition of microbial communities between the healthy individuals and the cases. Among the indices used, the most common alpha indices were Shannon, Chao1, and Simpson. In terms of beta diversity, the primary metrics utilized were Bray–Curtis dissimilarity, unweighted UniFrac distances, and weighted UniFrac distances. 

In the PD studies, a total of 16 studies showed no difference in alpha diversity between the PD cases and the controls [14,20,22,23,25,27,28,38,39,41,43,44,51,55,56,60]. However, eight studies reported distinct differences in gut microbiota between the PD cases and the healthy controls [23,47,49,50,52,53,62,66]. Among these eight studies, six found higher alpha diversity in the PD cases [23,49,52,53,62,66], while two observed lower alpha diversity in the cases compared to the controls [47,51] (Table 2). 

Regarding beta diversity analysis in the PD studies, consistent dissimilarities were found between the PD cases and the controls in 20 studies [19,20,21,22,23,24,25,38,39,41,44,47,49,50,52,53,56,60,62,66], indicating a notable divergence in gut microbiota composition. However, four studies did not identify any differences in beta diversity between the two groups [27,48,51,60].

In the AD studies, eight studies indicated no significant difference in alpha diversity between the AD cases and the controls [45,46,54,58,59,61,63,64]. Conversely, six studies demonstrated a lower alpha diversity in the AD cases compared to the controls [18,26,28,40,42,57], suggesting a potential alteration in the gut microbiota composition associated with AD.

In terms of beta diversity, 11 studies identified a difference in gut microbiota composition between the AD cases and the controls [17,18,28,40,42,45,54,57,59,61,63], implying distinct microbial community structures. However, five studies did not observe any dissimilarities in beta diversity between the two groups [44,46,57,58,65] (Table 2).

### 3.9. Gut Microbiota Associated with PD and AD (Differential Abundance Analysis (DAA))

A variety of statistical methods were utilized to identify differentially abundant bacteria between the cases and the controls in PD and AD studies. Among the commonly employed methods, seven studies used ANOSIM (analysis of similarities) [14,50,52,53,60,62], four studies used PERMANOVA (permutational multivariate analysis of variance) [24,49,55,56], LEfSe (linear discriminant analysis effect size) [21,27,47,66], and ANCOM (analysis of composition of microbiomes) [19,20,24,38] in the PD studies. In the AD studies, LEfSe was used in eight studies [28,42,45,46,54,57,59,63], while PERMANOVA was used in six studies [17,40,58,59,61,63].

It is worth noting that each method has its own strengths and considerations. Some studies may combine different methods to gain a comprehensive understanding of the differential abundance of bacterial taxa associated with PD and AD. Conversely, certain studies lacked sufficient details regarding these analyses.

The comparison of the DAA findings revealed several bacterial taxa that exhibited differential abundance between the cases and the control groups in the PD and AD studies. 

In the PD cases, differentially abundant bacteria were described as *Akkermansia* (seven studies) [20,44,47,49,51,52,56], *Lachnospiraceae* [21,22,24,41,51] and *Verrucomicrobiaceae* [19,20,24,47,56] (each in five studies), *Bifidobacterium* [44,50,56,60], and *Verrucomicrobia* (each in four studies) [14,21,49,56]. Other bacterial communities, such as *Ruminococcaceae* [21,47,60], *Actinobacteria* [56,60,66], *Akkermansia muciniphila* [19,38,52], *Bifidobacteriaceae* [24,27,56], *Bilophila* [38,44,49], *lactobacillus* [38,49,50], *Oscillospira* [14,22,50], *streptococcus* [38,48,56], and *Veillonella* [49,55,56], were reported in three studies each.

**Figure 5:**
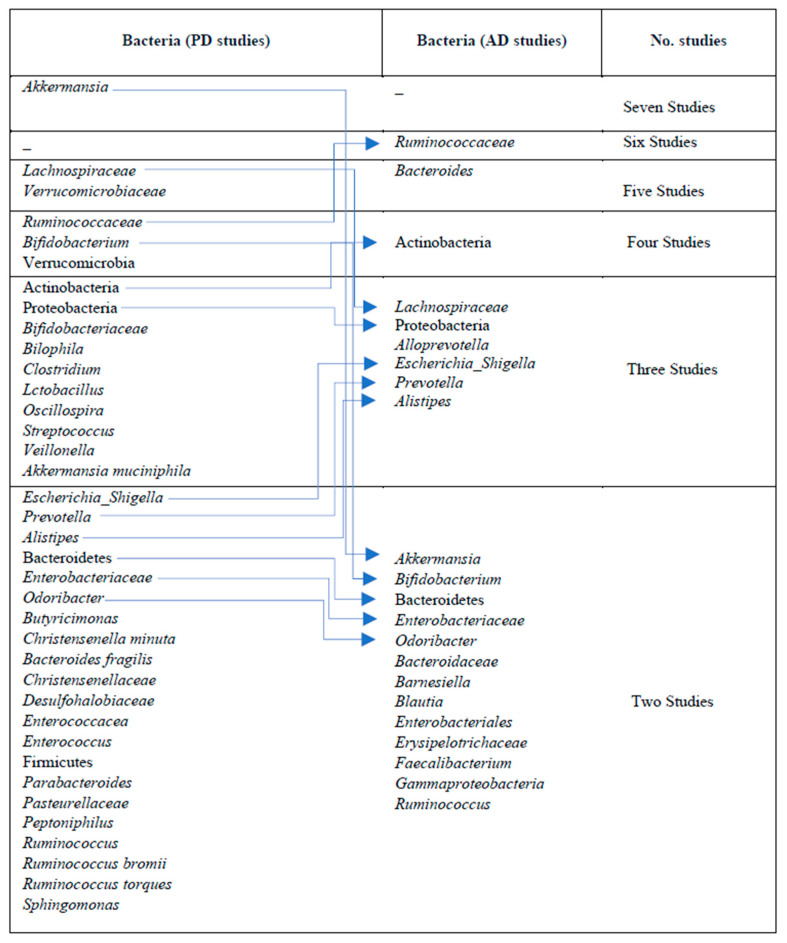
Differentially abundant microbial communities associated with PD and AD. Microbial communities identified in both PD and AD are shown using connecting arrows.

In the AD cases, the differentially abundant bacteria included *Ruminococcaceae* (six studies), *Bacteroides* (five studies), Actinobacteria (four studies), and *Alistipes*, Alloprevotella, *Escherichia*_*Shigella*, *Lachnospiraceae*, *Prevotella*, and Proteobacteria (each in three studies). There were also several bacteria identified in only one study. These bacteria have not been consistently reported across multiple studies but still may hold potential significance (Figure 5). 

In individuals with AD, certain microbial communities appear to be associated with potential mechanisms contributing to the disease: microbial communities such as *Bacteroides* and *Prevotella* may trigger inflammation and immune activation in the gut [67], while microbial groups like *Ruminococcaceae*, *Faecalibacterium*, *Butyricimonas*, and *Odoribacter* are associated with the production of metabolites, such as SCFAs, which can play a significant role in gut–brain communication [68]. Additionally, *A. muciniphila*, and *Verrucomicrobiaceae* are implicated in mucin degradation and the disruption of gut barrier integrity [69]. The diversity and balance of the gut microbiota may also play a role, with microbial communities like *Lachnospiraceae*, *Actinobacteria*, *Alistipes*, *Alloprevotella*, *Christensenellaceae*, and *Ruminococcus* contributing to this aspect. 

Also, in the PD cases, different microbial communities are associated with similar mechanisms. *Enterobacteriaceae*, *Escherichia_Shigella*, and *Gammaproteobacteria* may contribute to inflammation and immune activation, while *Bifidobacterium* and *Blautia* are linked to metabolite production. *Akkermansia* and *Verrucomicrobia* may be involved in mucin degradation and gut barrier function. The importance of comprehending the gut–brain connection in the context of neurodegenerative diseases may be highlighted by these shared mechanisms involving particular bacterial communities and metabolites. These mechanisms may also open up opportunities for therapeutic interventions that target the gut microbiota to reduce the risk and progression of both AD and PD.

## 4. Discussion

This systematic review aims to comprehensively evaluate the existing literature on gut microbiota composition in patients diagnosed with Parkinson’s and Alzheimer’s disease. 

In this study, the evaluation of the core gut microbiota observed in both the case and the healthy groups, irrespective of their health condition, showed the abundance of six main phyla, Bacteroidetes, Firmicutes, Proteobacteria, Actinobacteria, Verrucomicrobia, and Fusobacteria, and three families, *Ruminococcaceae*, *Bacteroidaceae*, and *Lachnospiraceae*, which also play a crucial role in maintaining the overall human health and gut homeostasis through several pathways, such as carbohydrate metabolism, short-chain fatty acid (SCFAs) production [70], regulation of immune response [71], intestinal barrier maintenance, and vitamin production [72,73]. 

Microbial taxa, such as *Akkermansia*, *Verrucomicrobiaceae*, *Lachnospiraceae*, *Ruminococcaceae*, *Bifidobacterium*, and *Proteobacteria*, among others, were found to be predominant in the PD cases. These bacterial communities are associated with various functions and metabolites that could potentially contribute to the pathology of PD. For instance, the capacity of *Akkermansia* (a member of the *Verrucomicrobiota phylum*) to degrade the mucus of the gut barrier can result in increased gut permeability (leaky gut). This condition can potentially enable the translocation of specific molecules, such as lipopolysaccharides (LPS), from the gut into the bloodstream, thereby initiating immune responses and systemic inflammation, which are implicated in neurodegenerative diseases. Also, it may impact the vagus nerve, a major pathway connecting the brain and the gut, and disturb the gut–brain axis communication [15]. The family *Lachnospiraceae* is also known to produce SCFAs, such as butyrate. Dysregulation of SCFAs may impact the gut–brain axis and contribute to neuroinflammation and PD progression [11].

Similarly, in the AD cases, *Acidobacteriota*, *Ruminococcaceae*, *Bacteroides*, *Proteobacteria*, and *Alistipes* appear as highly abundant bacteria. An imbalanced abundance of these bacterial taxa can have detrimental consequences for gut barrier function and can induce neuroinflammation through the production of metabolites and promote amyloid-beta aggregation, all of which contribute to the development and progression of AD [74,75].

Future studies should investigate these mechanisms in more detail to provide a clearer understanding of how the gut microbiota influences disease development and progression.

It is also interesting to note that some bacteria were observed in both the PD and the AD cases, such as *Alistipes*, *Bifidobacterium*, *Lachnospiraceae*, and *Proteobacteria*, although their abundance may differ. This overlap may highlight the possibility of shared interactions or mechanisms in the gut microbiota in these disorders. Further research is required to explore these shared aspects and determine whether common pathways or dysregulated processes contribute to the similarities observed in gut microbiota composition. 

The comparison of differentially abundant microbial taxa between the PD cases and the healthy controls, as well as the AD cases and the healthy controls, showed that the PD cases were enriched with *Akkermansia*, *Lachnospiraceae*, *Verrucomicrobiaceae*, *Bifidobacterium*, *Ruminococcacea*, Verrucomicrobia, Actinobacteria, and other bacterial taxa. Similarly, *Ruminococcaceae*, Actinobacteria, *Bacteroides*, *Alloprevotella*, *Escherichia/Shigella*, *Prevotella*, and Proteobacteria were differentially abundant between the AD cases and the AD controls. It is worth pointing out that although this association might suggest a microbial signature associated with PD and AD, it only indicates a correlation and does not establish a causal relationship between the identified bacterial taxa and the development of PD or AD. 

Furthermore, emerging studies on the gut–brain axis have highlighted bidirectional communication between the gut and the dopaminergic system, involving the intricate interaction of neurotransmitters like dopamine with the gut microbiota [76]. Dysbiosis has been associated with disruptions in these signaling pathways, potentially impacting the regulation of dopamine levels. Some bacterial species have been found to produce metabolites that can influence the dopaminergic system, while others may trigger inflammatory responses affecting the brain. The increased abundance of bacteria like *Ruminococcus*, *Enterobacteriaceae*, and *Clostridium*, among others, along with their metabolites, has been linked to dopamine dysregulation and the development of PD [77]. Understanding the role of gut microbiota dysbiosis and its impact on dopamine activity in neurological diseases may offer opportunities for diagnosis, prevention, and treatment.

Also, recent investigations on the association of nutrition, gut microbiota, and the development of neurodegenerative diseases have yielded interesting results. Although our primary objective in this systematic review did not include this aspect, it is important to recognize the significance of nutritional factors and potential mechanistic pathways when investigating the gut microbiota’s role in neurodegenerative diseases. Dietary choices, including the administration of probiotics (such as strains of *Lactobacillus* and *Bifidobacterium*) [78] have demonstrated their ability to influence the composition of the gut microbiota profile. Furthermore, certain dietary patterns, such as adherence to the Mediterranean diet [79] or increased dietary fiber intake [80], have shown associations with a more stable and beneficial microbial profile among individuals diagnosed with PD or AD. These findings have significant potential for the development of personalized nutrition interventions aimed at preventing these neurological conditions.

The evaluation of alpha diversity (microbial diversity within a given sample) and beta diversity (microbial diversity between groups) showed variable results across the different studies. In 24 studies (16 PD and 8 AD studies), no alpha diversity difference was observed between the cases and the controls. However, 14 studies reported distinct alpha diversity between the cases and the controls, with lower alpha diversity detected in the cases: (8 studies: 2 PD and 6 AD) and higher alpha diversity observed in 6 PD studies. Lower alpha diversity, indicating a less diverse gut microbiota, has been consistently linked to numerous diseases, such as irritable bowel syndrome (IBS) and fibromyalgia, along with other conditions [81,82]. Reduced alpha diversity is believed to influence the regulation of immune responses, metabolic processes, gut barrier function, and neurotransmitter function [71].

In terms of beta diversity, 31 studies (20 PD and 11 AD) demonstrated a distinct community structure between the cases and the controls, while 9 studies (4 PD and 5 AD) showed comparable community structures with a high degree of similarity between the two groups.

These findings highlight the heterogeneity in the gut microbiota composition in the PD and AD studies. It is also possible that the gut microbiota alterations in PD and AD are not universally consistent, reflecting the complex nature of these diseases and the potential involvement of multiple factors (such as study design, sample size, geographical location, and patient characteristics) in their pathogenesis [83]. Additional analyses, such as functional profiling of the microbiota or the investigation of specific bacterial taxa, metabolites, or functional pathways, can provide further insights into the potential mechanisms underlying the associations between the gut microbiota and PD or AD.

### 4.1. Limitation Described in Included Studies as Claimed by the Authors

1. Lack of covariate consideration: Important covariates such as diet, exercise, smoking, drug treatment, and comorbidities were not adequately addressed in some studies (13 studies).
2. Lack of longitudinal data: Several studies lacked longer follow-up periods to capture the microbial community changes during disease progression. Also, the cross-sectional design of many the studies limited their ability to establish causal relationships between the gut microbiota and neurodegenerative disorders (nine studies).
3. Small sample size: Several studies reported small sample sizes, which may have limited the statistical power and generalizability of the findings (six studies).
4. Lack of host–microbiome interaction consideration: The studies often did not consider the interactions between the host metabolism and the gut microbiota, which could provide a more comprehensive understanding of the mechanisms underlying neurodegenerative disorders (six studies).
5. Lack of species/strain resolution: The use of the 16S rRNA sequencing method limited the ability to analyze microbial composition at the species or strain level, which is crucial for identifying the specific microorganisms associated with the diseases (five studies).
6. Lack of mucosal microbiota analysis: Although nearly all of the included studies only utilized stool samples, only two studies acknowledged the need for the analysis of mucosal microbiota composition using gastrointestinal biopsies. Such analysis provides a deeper understanding of the local host–microbiota interaction (two studies).

As the authors of the included studies identified these limitations in their studies, these should be considered when interpreting the results. Addressing these limitations in future research can enhance the understanding of the gut microbiota’s role in neurodegenerative disorders and provide more robust insights into their underlying mechanisms.

### 4.2. Strength and Limitation of This Systematic Review

This systematic review has several strengths. First, it was registered with PROSPERO, which highlights its transparency and adherence to pre-established protocols. Additionally, it includes the highest number of studies to date, enhancing the comprehensiveness and interpretation power of the review. It also involves the extraction and comparison of significant findings related to the gut microbiota in relation to PD and AD.

It is important to note that while bacteria are the predominant community in the gut, other forms of intestinal flora, such as mycobiota, archaeome, protozoa, and virome, also exist, although in lesser amounts. However, due to insufficient research on these communities, they were not considered in this review, thus representing a limitation of the study.

### 4.3. Future Recommendations

1: Considering important covariates such as diet, exercise, smoking, comorbidities, and drug treatment and their potential influence on the gut microbiota as a fundamental step in the study design would provide a more comprehensive insight into the role of gut microbiota in AD and PD diseases.
2: The design of longitudinal studies with longer follow-up periods to capture microbial community changes during disease progression is needed. Also, well-designed intervention studies, such as probiotic or prebiotic trials, can help to determine the therapeutic potential of modulating the gut microbiota in relation to disease symptoms and progression.
3: Large-scale cohort studies involving diverse populations with larger sample sizes and frequent sampling to capture variations in gut microbiota composition associated with different backgrounds, geographical locations, and lifestyles are essential. This will help to identify potential factors influencing gut microbiota and allow for personalized approaches to managing diseases using gut microbiota markers. Furthermore, considering the heterogeneity among ethnic groups, which is reflected in the wide variation in microbiota, categorizing research studies according to their geographical location and subsequently comparing outcomes between regions could provide a valuable basis for a deeper understanding of gut microbiota profile in diverse human populations.
4: Investigating the functional analysis of gut microbiota by exploring metabolomic and metagenomic approaches can provide insights into specific mechanisms underlying disease pathogenesis. Also, utilizing advanced sequencing techniques such as shotgun metagenomics allows species- and strain-level resolution, and other omics approaches, such as metratranscriptmics and metabolomics, allow the understanding of the mechanistic insight into host–microbiota interactions.
5: Additionally, incorporating sigmoid mucosal biopsies and detailed characterization of microbial functions would enhance the understanding of host–microbiota interactions.
6: Establishing standardized microbiota protocols from sample collection to data analysis to enhance the reliability and comparability of microbiota findings would lead to a better understanding of the dynamic relationship between the host and the gut microbiota [84].

## 5. Conclusions

In conclusion, this systematic review provided evidence indicating a link between gut microbiota dysbiosis and PD and AD. Additionally, it is important to consider the limitations of the included studies and to note that the findings have been inconsistent, suggesting that a single microbial community or factor may not fully explain the complexities of these diseases. Instead, it is likely that a network of microbial communities, along with multiple host and environmental factors, contribute to the development and progression of these disorders. Further research is needed to evaluate the host–gut microbiota interactions more effectively to provide novel personalized therapeutic interventions and preventive strategies targeting the gut–brain axis more effectively.

## Figures and Tables

**Table 1:** Summary of included studies (see Supplementary Material S4 for complete data extraction table).

Study	Year	Location	Population Size	Age	Gender	Sample Type	Matching Factor	Sample Preservative	Extraction Method	Sequencing Platform	Sequenced Regions	Forward Primer	Reverse Primer	Data Availability	Main Microbiota Finding
[38]	2019	Finland	128 (64 case, 64 control)	case: 65.2 ± 5.52/control: 64.45 ± 6.9; (years, mean ±SD)	Female: case: 48.6%/control: 50.0%	stool	age, sex	DNA stabilizer PSP (Spin Stool DNA Plus Kit, STRATEC Molecular)	PSP Spin Stool DNA Plus Kit (STRATEC Molecular)	Illumina MiSeq	V3–V4	341F1–4 (5′ CCTACGGGNGGCWGCAG 3′)	785R1–4 (5′ GACTACHVGGGTATCTAATCC 3′)	PRJEB27564	Significant differences in gut microbiota between cases and controls. Disease progression did not influence gut microbiota. No difference in Firmicutes/Bacteroidetes ratio between cases and controls.
[39]	2020	Ireland	309 (147 case, 162 control)	case: 69.3 ± 8.6/control: 63.3 ± 8.3; (mean ± SD)	Female: case: 31.5%/control: 35.8%	stool	age, sex	MNIgene.GUT^®^ kit	Chemagic DNA blood protocol	Illumina MiSeq	V3–V4	NR	NR	Available on request	Composition of the gut microbiome could potentially serve as a marker of disease severity in PD. *Bilophila* and *Paraprevotella* abundance were significantly associated with disease severity.
[23]	2018	Italy	350 (237 cases, 113 control)	cases: 67.6 (9.7)/control: 65.9 (9.9); (y, mean (SD))	cases: 115 (59.6)/control 47 (41.6): Male gender, *n* (%)	stool	age, nutritional status, geographical area	No preservative	QIAamp DNA Stool Mini Kit	Illumina MiSeq	V3–V4	NR	NR	NR	Low *Lachnospiraceae* in cases.
[19]	2017	Germany	59 (31 case, 28 control)	case: 64.8 ± 9.5/control 65.6 ± 10.4; (years, mean ± SD)	Male: all case and control	stool	age	NR	NR	Illumina Hiseq4000	NR	NR	NR	ERP019674	Case and controls had different gut microbiota composition, characterized by increased levels of *Verrucomicrobiaceae* (*Akkermansia muciniphila*) and unclassified Firmicutes, and decreased levels of *Prevotellaceae* (*Prevotella copri*) and *Erysipelotrichaceae* (*Eubacterium biforme*) in cases.
[44]	2020	Canada	300 (197 case, 103 control)	case:66 (59–71)/control 66 (58–71); (years)	Female: case: 38.1%/control: 51.5%	feces	age	DNA stabilizer buffer	OMNIgeneGUT Kit	Illumina MiSeq	V4	515F	806R	On publisher’s website	Increased abundance of *Akkermansia* and *Bifidobacterium* and decreased abundance of *Faecalibacterium*, *Lachnospiraceae*, and SCFA-producing bacteria in cases.
[22]	2020	Germany	101 (71 case, 30 control)	case: 65.3 ± 10.2/controls: 64.3 ± 8.9; (years)	Female: case 45.7%/controls: 45.2%	feces	MS score, NMS constipation item, Wexner Constipation, coffee consumption	DNA stabilizing solution	PSP R Spin Stool DNA *Plus* Kit	Illumina MiSeq	V3–V4	341F (5′-ACTCCTACGGGAGGCAGCAG-3′)	806R (5′-GGACTAC HVGGGTWTCTAAT-3′)	Available on request	High abundance of *Faecalibacterium*, *Ruminococcus*, *Clostridia*, *Lachnospiraceae*, *Oscillospira*, *Betaproteaobacteria*, *Burkholderiales*, *Alcaligenaceae*, and *Sutterella* in cases compared to controls.
[20]	2017	Germany	154 (76 case, 78 control)	case: 68.0 6 9.7/control: 68.4 6 6.7; (year)	Male: case 66%/control 59%	stool	diabetes medication	Stool specimen collector (MedAuxil)	Qiagen AllPrep kit	Illumina MiSeq	V4	515F	805R	PRJNA381395	High abundance of Akkermansia in PD, different gut microbiota between cases and controls.
[24]	2017	USA	327 (197 case, 130 control)	Total 69, 68.4, 9.2; (Median, Mean, SD)	Female: 65; 132 (67.0%), Male (% Male)	stool	PD medications, disease duration, spousal relationship, geographic site	Media-free swabs kit with DNA/RNA-free sterile swabs	According to the Earth Microbiome Project Protocol	Illumina MiSeq	V4	515F	806R	ERP016332	Significantly higher abundance of *Bifidobacteriaceae*, *Christensenellaceae*, *Lactobacillaceae*, *Pasteurellaceae*, and *Verrucomicrobiaceae* families in cases compared to controls/association of a high abundance of *Akkermansia*, *Lactobacillus*, *Bifidobacterium*, and reduced *Lachnospiraceae* (chain fatty acids producer) with disease development.
[41]	2017	Germany	58 (29 case, 29 control)	case: 69.2, 6.5/control: 69.4, 6.7 (years; mean, SD)	case: 23 male, 6 female/control: 13 male, 16 female	stool	age	Shipment with no preservative, at room temp	PowerSoil Kit	Illumina MiSeq	V1–V2	NR	NR	NR	High abundance of *Lactobacillaceae*, *Barnesiellaceae*, and *Enterococcacea* in cases.
[14]	2015	USA	72 (38 case, 34 control)	case: 61.6 ± 9.4/control: 45.1 ± 14.4; (mean)	case: 24/14, control: 18/16; male/female	fecal samples and sigmoid mucosal biopsies	NR	Supporting info	Supporting info	NR	V4	NR	NR	NR	Anti-inflammatory butyrate- producing bacteria from the genera *Blautia*, *Coprococcus*, and *Roseburia* were significantly more abundant in the feces of controls than PD patients/*Faecalibacterium* were significantly more abundant in the mucosa of controls than in PD/proinflammatory bacteria such as *Proteobacteria* were significantly more abundant in the mucosa of PD than controls/the ratio of Firmicutes-to-Bacteroidetes in fecal samples was not significantly different between PD and HC groups/positive correlation of *Bacteroidetes* and Proteobacteria with PD duration/negative correlation of Firmicutes with PD duration.
[47]	2019	China	99 (51 case, 48 control)	case: 62.4 ± 8.2, control: 62.2 ± 9.2 (years, mean ± SD)	case: male: 32, female: 19/control: control: male: 19, female: 29	stool	age	Sterilized tube	QIAamp Fast DNA Stool Mini Kit	HiSeq2500 PE250	V4	515F	806R	NR	Low alpha and beta diversity, high abundance of *Akkermansia*, *P. copri Prevotella*, *Ruminococcaceae*, *Veillonellaceae Verrucomicrobiaceae*, *methanobrevibacter smithii*, *Ruminococcus callidus*, *Roseburia inulinivorans*, *Parabacteroides merde*, *Ruminococcus torques* in cases and a high abundance of *Bacteroidales*, *Lactobacillaceae*, *lactobacillus gasseri* in controls.
[21]	2019	China	20 (10 case, 10 control)	over 65	case: male: 7 (70%)/control: male: 5 (50%)	feces	NR	DNA/RNA Shield	PSP SPIN Stool DNA plus kit	Illumina MiSeq	V1–V3	341F	805R	NR	Slightly different gut microbiota between cases and controls, a significant abundance of *Bacteroides* and *Prevotellaceae* in healthy controls, a significant abundance of *Ruminococcaceae*, *Verrucomicrobiaceae*, *Porphyromondaceae*, *Hydrogenoanaerobacterium*, and *Lachnospiraceae* in PD cases.
[27]	2018	China	120 (75 case, 45 control)	case: 60.48 ± 10.72/control; 63.20 ± 6.00	case: male: 49, female: 26/control: male: 23, female: 22	stool	age	Without preservation solution at room temperature during shipment	Huirui.^®^ DNA kit	Illumina HiSeq PE250	V4	V4T9 (5′-GTGTGYCAGCMG-CCGCGG TAA-3′)	V4R19 (5′-CCGGACTACNVGGGTWTCTAAT-3′)	NR	Significant reductions in *Tenericutes*, *Euryarchaeota*, and Firmicutes, *Lachnospiraceae* in patients with PD. *Veillonellaceae* and *Verrucomicrobiaceae* showed marked increases but without statistical significance. Significant differences in alpha diversity (but not beta) between patients with PD who had a disease duration of greater than 5 years compared to those with a disease duration of fewer than 5 years.
[49]	2019	Taiwan	157 (80 case, 77 control)	case: 62.3 ± 7.8/control: 61.8 ± 8.3	case: 62.5% men/control: 60% men	stool	age and sex	Flash-frozen on dry ice, and stored at −80 °C	QIAamp Fast DNA Stool Mini Kit	Illumina MiSeq	V3–V4	NR	NR	NR	Microbiota from patients with PD dominated by *Verrucomicrobia*, *Mucispirillum*, *Porphyromonas*, *Lactobacillus*, and *Parabacteroides*. In contrast, *Prevotella* was more abundant in controls. The abundances of *Bacteroides* were more increased in patients with non-tremor PD subtype than patients with tremor subtype. *Bacteroides* abundance was correlated with motor symptom severity defined by UPDRS part III motor scores.
[48]	2017	China	38 (24 case, 14 control)	case: 73.75 ± 6.26/control: 74.64 ± 5.57	case: 16 men/control: 6 men	stool	age and sex	NR	TIANamp stool DNA kit (Tiangen Biotech Co., Ltd., Beijing, China)	Illumina MiSeq	V3–V5	5′-CCTACGGRRBGCASCAGKVRVGAAT-3′	e 5′-GGACTACNVGGGTWTCTAATCC-3′	NR	The genera *Blautia*, *Faecalibacterium*, and *Ruminococcus* were significantly decreased in PD compared to healthy controls. The genera *Escherichia*-*Shigella*, *Streptococcus*, *Proteus*, and *Enterococcus* were significantly increased in PD subjects.
[50]	2017	Russia	155 (89 case, 66 control)	case: 67/control: 63	NR	stool	age and sex	NR	NR	Illumina MiSeq	V3–V4	NR	NR	NR	Reduction in taxonomic diversity of gut microbiota in patients with PD.
[51]	2019	Italy	152 (80 case, 72 control)	NR	NR	stool	age, sex, loss of weight	NR	PSP Spin Stool DNA Kit Plus (Stratec Molecular)	Illumina MiSeq	V3–V4	NR	NR	PRJNA510730	Significantly higher levels of *Lactobacillaceae*, *Enterobacteriaceae*, and *Enterococcaceae* families compared to healthy individuals. On the other hand, the levels of Lachnospiraceae were significantly reduced in PD patients.
[52]	2020	China	80 (40 case, 40 control)	case: 66.6 ± 7.1/control: 66.3 ± 8.1	case: 19 men/control: 21 men	stool	lifestyle, gender, age, and BMI	NR	QIAamp DNA Stool Mini Kit (Qiagen)	Illumina HiSeq X-ten	NR	NR	NR	PRJNA433459	The diversity and community of gut microbial genes in PD patients differed from those of healthy control subjects. Thirty-six different taxa were enriched in the PD patients, and no taxon was enriched in the healthy controls.
[53]	2018	China	90 (45 case, 45 control)	case: 68.1 ± 8.0/control: 67.9 ± 8.0	case: 22 men/control: 23 men	stool	age, sex, BMI, constipation	NR	QIAamp DNA Stool Mini Kit (Qiagen)	Illumina MiSeq	V3–V4	341F	806R	PRJNA391524	Some bacteria were correlated with PD clinical characteristics, including disease duration, severity, medication, and non-motor symptoms.
[66]	2020	China	40 (27 case, 13 control)	case: 62.1 ± 10.2/control: 63 ± 8.76	case: 19 men/control: 3 men	stool	age	NR	QIAamp DNA Stool Mini Kit (Qiagen)	Illumina MiSeq	V3–V4	515F 5′-GTGCCAGCMGCCGCGGTAA-3′	926R 5′-CCGTCAATTCMTTTGAGTTT-3′	PRJNA561023	Compared with HC and patients with PD-NC, the gut microbiota of patients with PD-MCI was significantly altered, particularly manifesting in enriched genera from *Porphyromonadaceae* family and decreased abundance of genera *Blautia* and *Ruminococcus*.
[55]	2021	Malaysia	200 (104 case, 96 control)	case: 65.4 ± 8.4/control: 62.4 ± 9.0	case: 62.5% male/control: 37.5% male	stool	age, sex	Preservatives were not added	QIAamp DNA Stool Mini Kit (Qiagen)	Illumina MiSeq	V3–V4	341F (5′-CCTACGGGNGGCWGCAG-3′)	805R (5′-GACTACHVGGGTATCTAATCC-3′)	PRJNA494620	Ten bacterial taxa were significantly increased in PD; largest fold changes were observed for *B. fragilis*, *Lactobacillus acidophilus*, *Megasphaera* and *Gammaproteobacteria*.
[56]	2020	Italy	115 (64 case, 51 control)	case: 71.39 ± 10.99/51.67 ± 12.42	case: 44 men/control: 31 men	stool	sex, age, BMI, coffee consumption, smoking	NR	QIAamp DNA Stool Mini Kit (Qiagen)	Illumina MiSeq	V3–V4	NR	NR	PRJEB30401	The most significant changes within the PD group highlighted a reduction in bacterial taxa, which are linked to anti-inflammatory/neuroprotective effects, particularly in the *Lachnospiraceae* family and key members, such as *Butyrivibrio*, *Pseudobutyrivibrio*, *Coprococcus*, and *Blautia*.
[43]	2019	Germany	59 (34 case, 25 control)	case: 67.9 ± 8.6/63.9 ± 5.8	case: 23 men/control: 11 men	stool	age	Sterile containers	FastDNA SPIN kit	Illumina MiSeq	V4–V5	520 F (5′-AYTGGGYDTAAAGNG-3′)	926 R (5′-CCGTCAATTCMTTTRAGTTT-3′)	PRJEB30615	PD patients exhibit alterations in their gut microbiota composition, characterized by a decrease in beneficial bacteria and an increase in certain bacterial groups. A potential link between the gut microbiome and PD development, as well as the influence of PD medications on the gut microbiota.
[60]	2021	China	40 (20 case, 20 control)	case: 63.65 ± 5.64/61.95 ± 4.73	case: 10 men/control: 10 men	stool	age, sex, BMI	NR	DNeasy PowerSoil Kit (Qiagen)	Illumina MiSeq	V3–V4	343 F: 5′-TACGGRAGGCAGCAG-3′	798 R: 5′-AGGGTATCTAATCCT-3′	NR	A greater abundance of *Alistipes*, *Rikenellaceae_RC9*_gut_group, *Bifidobacterium*, *Parabacteroides*, while *Faecalibacterium* was decreased in fecal samples from PD patients.
[62]	2020	China	126 (63 case, 63 control)	case: 64.0 ± 7.4/63.9 ± 7.9	case: 40 men/control: 23 men	stool	age	NR	QIAamp Fast DNA Stool Mini Kit (Qiagen)	Illumina HiSeq PE250	V4	NR	NR	CRA001938	There were markedly different microbial compositions among PD, HS, and HP samples by alpha/beta diversity. They also found differential microbial compositions among Hoehn and Yahr stage/disease duration.
[25]	2014	Finland	144 (72 case, 72 control)	case: 65.3 ± 6 5.5/64.5 ± 6.9	case: 51.4% male/control: 50% men	stool	age, sex	NR	NR	NR	V1–V3	NR	NR	NR	Reduced abundance of *Prevotellaceae* in PD patients and the positive association of *Enterobacteriaceae* abundance with PIGD symptoms.
[40]	2022	China	172 (132 case (mild: 43, moderate: 89), 40 control)	60 to 90	M/F case: 15/28 (mild AD), 33/56 (moderate AD)/control: 16/24	feces	age, sex	Special cytoprotective agent	E.Z.N.A. Soil DNA Kit	Illumina MiSeq	V3–V4	CCTACGGGNGGCWGCAG	GACTACHVGGGTATCTAATCC	PRJNA855571	Elevated abundance of certain bacteria in cases (moderate vs. control), including *Proteobacteria*, *Verrucomicrobia*, *Actinobacteria*, and *Synergistetes*. Reduced levels of Firmicutes and *Bacteroidetes* in cases. Controls, when compared to mild and moderate cases, showed higher levels of *Firmicutes*, *Erysipelotrichia*, *Acidaminococcaceae*, *Ruminococcaceae*, and *Bacteroidetes*.
[26]	2022	Canada	99 (45 case, 54 control)	case: 74: (65–78)/control: 70 (66–74); (year)	Female: case 33.3%/control 33.3%	feces	age	OMNIgene^®^ GUT	QIAamp PowerFecal DNA Kits	MiSeq	V4	515F (GTGCCAGC MGCCGCGGTAA)	806R (GGACTACHVHHHTWTCTAAT)	PRJNA770746	No significant different between AD patients and controls (beta diversity), lower alpha diversity in cases, higher abundance of *Erysipelotrichaceae* in cases.
[45]	2021	China	64 (28 cases (18 AD), 18 control)	case: 63.5 (4.7)/control: 64.5 (4.5); (SD)	Male (%): case 2 (11)/control: 4 (22)	feces	age, sex	NR	PowerSoil DNA Isolation Kit	Illumina Miseq/Microseq	V3–V4	NR	NR	NR	AD cases exhibited increased microbial diversity, decreased levels of *Bacteroides*, *Lachnospira*, and *Ruminiclostridium*, and increased *Prevotella.*
[17]	2019	USA	108	case: 84.7 (8.1)/control: 83 (10.2); (mean [SD]) (year)	Male: case: 4 (16.7)/control: 8 (15.7)	stool	age, sex	NR	PowerMag soil DNA isolation kit	NextSeq 500	NR	NR	NR	Upon request	Significant increase in *Bacteroides*, *Alistipes*, *Odoribacter*, *Barnesiella*, *Osplanchnicus*, *Odoribacter* spp., *K. pneumoniae*, *B. fragilis*, *E. lenta*, and *Desulfovibrio* AD (sulfate-reducing bacteria). Conversely, there were significant decreases in bacteria, including *Lachnoclostridium*, *Butyrivibrio*, *B. proteoclasticus*, *B. hungatei*, *Eubacterium*, *E. eligens*, *E. hallii*, *E. rectale*, *Clostridium* sp. *SY8519*, *R. hominis*, and *F. prausnitzii.*/significantly different beta diversity between controls and AD cases.
[46]	2022	Kazakhstan	84 (41 case, 43 control)	case: 68 (62–74)/control 68 (61–75); (median (IQR))	Female, *n* (%): case 30 (73.2%)/control 35 (81.4%)	feces	NR	special kit	QIAamp DNA stool Mini Kit	Illumina MiSeq	NR	NR	NR	PRJNA811324	Increased abundance of *Acidobacteriota*, *Verrucomicrobiota*, *Planctomycetota*, and *Synergistota* and decreased abundance of *Bifidobacterium*, *Clostridia* bacterium, *Castellaniella*, *Erysipelotrichaceae UCG-003*, *Roseburia*, *Tuzzerella*, *Lactobacillaceae* and *Monoglobus* in AD patients.
[28]	2019	China	97 (33 case (32 aMCI), 32 control)	case: 74.85 ± 11.37 (AD), 70.53 ± 11.0 (aMCI)/control: 76.88 ± 9.35; (years, mean ± SD)	Male: case: 14 (AD; 42.42%), 18 (56.25%) aMCI/control 16 (50%)	feces	age, sex	Sterile collection containers	QIAGEN	Illumina MiSeq	V3–V4	5′-CAAGCAGAAG ACGGCATACGAGATGTGACTGGAGTTCAGACGTGTGCTCTTCCGA TCT-3′	5′-AATGATACGGCGACCACCGAGATC TACACTCTTTCCCTACACGACGCTCTTCCGATCT-3′	PRJNA496408	Decreased diversity in AD patients compared to controls. Different gut microbiota between healthy and cases/reduced abundance of Firmicutes, increased abundance of *Proteobacteria* in cases/a significant correlation between AD severity and the abundance of altered microbiomes/association of *Enterobacteriaceae* with AD.
[42]	2021	China	105 (case: 53 (SCD) 14 (CI: MCI, *n* = 8; mild AD dementia, *n* = 6))/38 control)	case: 66.68 ± 6.32 (SCD), 73.21 ± 7.89 (CI)/control: 66.79 ± 5.13; (year)	M/F: case: 10/43 (SCD), 4/10 (CI)/control: 15/23	feces	age, sex, educational years, other potential factors	Cytoprotective agents	QIAamp DNA Stool Mini Kit	Illumina Miseq PE250	V3–V4	341F (CCTAC GGGRSGCAGCAG)	806R (GGACTACVVGGGTATCTAATC)	NR	Decreasing abundance of Firmicutes, *Clostridia*, *Clostridiales*, *Ruminococcaceae*, and *Faecalibacterium* in cases.
[54]	2020	Austria	41 (23 case, 18 control)	case: 88 (73, 85)/control: 75 (74, 76); (years)	F/M: case: 15/18, control: 11/7	stool	age, sex	Collection tubes	MagnaPure LC DNA Isolation Kit III	Illumina MiSeq	V1–V2	27F (AGAGTT TGATCCTGGCTCAG)	R357 (CTGCTGCCTYCCGTA)	PRJNA608281	Decreased abundance of *Lachnospiraceae* in cases, clear clustering of case and controls at different stages of dementia according to beta diversity, AD association with a reduction in bacteria producing short-chain fatty acids (SCFA) and increased biomarkers of gut permeability and inflammation. Increased abundance of *C. clostridioforme* and *Eisenbergiella* is associated with cognitive impairment.
[18]	2022	Spain	22 (12 case, 10 control)	60 to 70	case: 2 men/control: 6 men	stool	NR	NR	QIAamp PowerFecal Pro DNA isolation kit (Qiagen, Madrid, Spain)	Illumina MiSeq	NR	515F-Y (50 GTG YCA GCM GCC GCG GTA A 30	806R (50 GGA CTA CNV GGG TWT CTA AT 30	NR	At a more advanced stage of AD, the gut microbiota and volatiles shifted towards a profile with increases in *Ruminococcus* and *Blautia.*
[58]	2022	Thailand	40 (20 case, 20 control)	case: 72.8 ± 5.6/control: 69.4 ± 6.2	case: 45.5% male/control: 38.5% male	stool	NR	Preservation System (Norgen Biotek Corp., Thorold, ON, Canada)	QIAamp Stool Mini kit (Qiagen, USA)	Illumina MiSeq	V3–V4	NR	NR	NR	A significant difference at the operational taxonomic unit level. The altered gut microbiome could be potentially targeted for the early diagnosis of dementia and the reduction in AD risk.
[59]	2021	China	65 (21 case, 44 control)	case: 76.2 ± 9.9/control: 78.4 ± 6.6	case: 13 men/control: 20 men	stool	NR	Sterile fecal collection containers	E.Z.N.A.@ Stool DNA Kit (Omega Bio-Tek, Norcross, GA, USA)	Illumina Miseq	V3–V4	338 forward (5′-ACTCCTACGGGAGGCAGCAG-3′)	806 reverse (5′-GGACTACHVGGGTWTCTAAT-3′)	SRP252374	Gut microbial alterations and related metabolic output changes may be associated with pathogenesis of AD. Fecal markers might be used as a non-invasive examination to assist screening and diagnosis of AD.
[61]	2022	Turkey	98 (47 case, 51 control)	case: 71.4 ± 5.1/control: 67 ± 5.3	case: 24 men/control: 28 men	stool	NR	NR	QiaAmp DNA stool minikit (Qiagen, Germany)	Illumina MiSeq	V3–V4	341 F (59-CCTACGGGNGGCWGCAG-39)	805 R (59-GACTACHVGGGTATCTAATCC-39)	NCBI BioProject database	A different gut microbiota composition in AD cases marked primarily by *Prevotella* and *Bacteroides*, but also subnetworks of other taxa exist in the community.
[63]	2021	China	92 (60 case, 32 control)	case: 72.82 ± 7.25/control: 71.06 ± 5.92	case: 24 men/control: 14 men	stool	age	NR	QIAamp^®^ DNA Stool Mini Kit (Qiagen, Hilden, Germany)	Illumina MiSeq	V3–V4	5-CCTACGGGNGGCWGCAG-3	5-GACTACHVGGGTATCTAATCC-3	NR	AD patients had gut microbiota alterations related to cognition, and differential taxa between AD patients with and without NPS associated differently with NPS domains.
[65]	2018	China	86 (43 case, 43 control)	case: 70.12 ± 8.76/control: 69.72 ± 9.24	case: 23 men/control: 23 men	stool	age, sex	NR	QIAamp^®^ DNA Stool Mini Kit (Qiagen, Hilden, Germany)	Illumina MiSeq	V3–V4	338F	806R	NR	Altered gut microbiota composition and diversity AD cases compared to cognitively normal controls. Several bacterial taxa, including *Actinobacteria*, *Bacteroidales*, *Ruminococcaceae*, *Selenomonadales*, and *Lachnoclostridium*, were found to contribute to these differences.
[64]	2022	China	302 (125 MCI, 83 AD case, 94 control)	case: 71.8 ± 8.3/control: 74.3 ± 10.6	case: 53 men/control: 58 men	stool	NR	Sterile containers	E.Z.N.A.^®^ soil DNA Kit (Omega Bio-tek, Norcross, GA, USA)	Illumina MiSeq	V3–V4	338F (50 -ACTCCTACGGGAGGCAGCAG-30)	806R (50-GGACTACHV GGGTWTCTAAT-30)	NR	No significant difference in the alpha and beta diversity among groups. Patients with AD or MCI had increased bacterial taxa including *Erysipelatoclostridiaceae*, *Erysipelotrichales*, *Patescibacteria*, *Saccharimonadales*, and *Saccharimonadia*, compared with NC group.
[57]	2017	USA	50 (25 case, 25 control)	case: 71.3 ± 7.3/control: 69.3 ± 7.5	case: 8 men/control: 7 men	stool	age, sex	NR	NR	Illumina MiSeq	V4	NR	NR	NR	Decreased microbial diversity in AD cases and compositionally distinct from controls.

**Table 2:** Summary of alpha and beta diversity findings between cases and controls in PD and AD studies.

	PD Studies	AD Studies
**Alpha Diversity (case vs. control)**	No difference observed (16 studies) Difference observed (8 studies) - 6 studies higher alpha diversity in cases - 2 studies lower alpha diversity in cases	No difference observed (8 studies) Difference observed (6 studies) - 6 studies lower alpha diversity in cases
**Beta Diversity (case vs. control)**	No difference observed (4 studies) Difference observed (20 studies)	No difference observed (5 studies) Difference observed (11 studies)

## Data Availability

All relevant data are within the manuscript and Supplementary Materials.

## Supplementary Materials

The following supporting information can be downloaded at: https://www.mdpi.com/article/10.3390/nu15204365/s1.
