# Supplementary material for: Gut Microbiota Composition in Patients with Neurodegenerative Disorders (Parkinson’s and Alzheimer’s) and Healthy Controls: A Systematic Review
Source: Nutrients. 2023 Oct 13;15(20):4365. doi: 10.3390/nu15204365 (PMC10609969; doi:10.3390/nu15204365)
Supplement: Supplementary file 1 [file nutrients-15-04365-s001.zip › 1_Supp_SearchingStrategy.pdf]

Search Strategy:

Relevant and comprehensive biomedical research databases (PubMed, Medline, and Embase) were searched without language, date, or location restriction, following the Preferred Reporting Items for Systematic Reviews and Meta-analyses guideline (PRISMA) (Moher D, Shamseer L, Clarke M, Ghersi D, Liberati A, Petticrew M, Shekelle P, Stewart LA. Preferred reporting items for systematic review and meta-analysis protocols (PRISMA-P) 2015 statement. Systematic reviews. 2015 Dec;4(1):1-9.

Search terms:

To retain the highest number of hits, the following search terms were selected in combination with different logical operators (AND,OR,(), "",*): (((((((microbiome)) OR (microbiota)) OR (intestinal flora) OR (intestinal microbi*) OR (dysbiosis) OR (gut microbio*)) AND (Parkinson's Disease)) OR (Alzheimer's Disease)

Filter 1: Non-English manuscript

Filter 2: Non-Human studies

Filter 3: Reviews studies

PubMed > (https://pubmed.ncbi.nlm.nih.gov/advanced/)

Embase > (https://ovidsp.dc2.ovid.com/ovid-a/ovidweb.cgi?ID=shib%3Adc1%3A0x4f50f21f1be34bb29cdecbbbc99b6b86&PASSWORD=0x4f50f21f1be34bb29cdecbbbc99b6b86&CSC=Y&T=JS&D=emcz&NEWS=n&PAGE=main&entityID=https%3A%2F%2Fidp.unimelb.edu.au%2Fopenathens)

Medline > (https://ovidsp.dc2.ovid.com/ovid-a/ovidweb.cgi?ID=shib%3Adc1%3A0xc98a08360f1a48b5b16c7db10917e639&PASSWORD=0xc98a08360f1a48b5b16c7db10917e639&CSC=Y&T=JS&D=ppez&NEWS=n&PAGE=main&entityID=https%3A%2F%2Fidp.unimelb.edu.au%2Fopenathens)

Search result:

**Searching Studies**	**PubMed**	**Embase**	**Medline**	**Total**
No Filter	197715	6325	10002	214042
Filter 1: Non-English manuscript	188988	6160	9836	204984
Filter 2: Non-Human studies	137833	4282	5141	147256
Filter 3: Review studies	5079	203	2645	7927
Filter 4: Full-text availability	5016	24	587	5627
Total before removing duplicates	5627			
Number of duplicate studies	21			
Total after removing duplicates	5606			

Inclusion criteria:

- Human studies
- Studies comparing the gut microbiota composition between healthy individuals and Parkinson's or Alzheimer's patients.

- English articles
- Molecular-based approaches (including 16S rRNA sequencing or shutgun metagenomic studies)

Exclusion criteria

- Animal studies
- Studies without appropriate healthy and control groups
- Non-English articles
- Studies with inappropriate method used including culture-bases studies or targeted PCR
- Review papers
- Duplicates
