# Supplementary figures and images for: Gut Microbiota Composition in Patients with Neurodegenerative Disorders (Parkinson’s and Alzheimer’s) and Healthy Controls: A Systematic Review
Source: Nutrients. 2023 Oct 13;15(20):4365. doi: 10.3390/nu15204365 (PMC10609969; doi:10.3390/nu15204365)

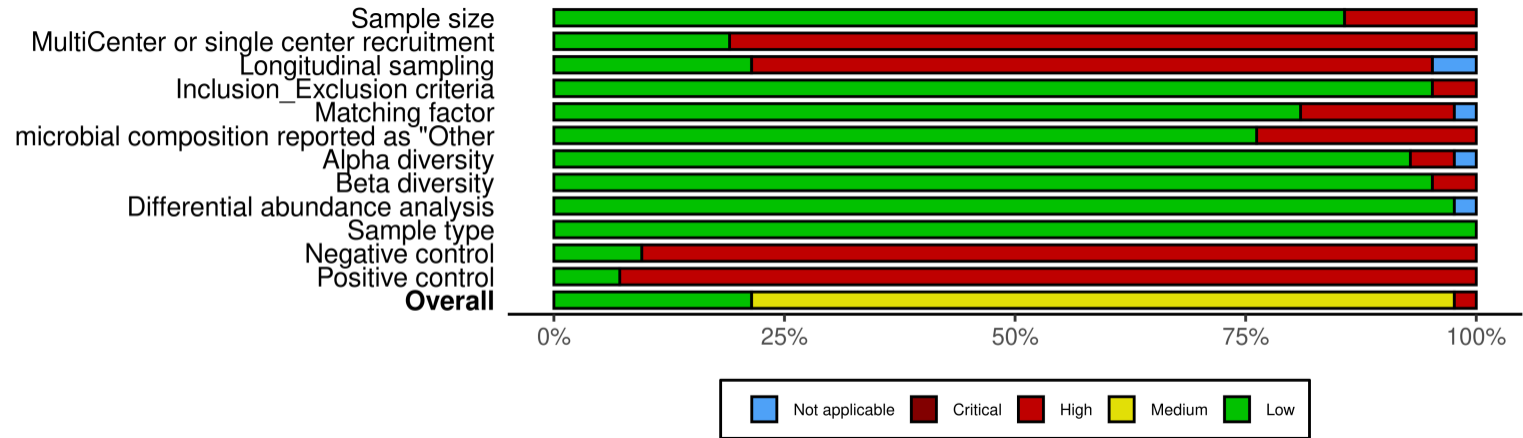

Supplement: Supplementary file 1 [file nutrients-15-04365-s001.zip › 3_Bias-Summary-Plot.pdf]
